# Supplementary material for: Precision engineering of biological function with large-scale measurements and machine learning
Source: PLoS One. 2023 Mar 29;18(3):e0283548. doi: 10.1371/journal.pone.0283548 (PMC10057847; doi:10.1371/journal.pone.0283548)
Supplement: Attachment — Submitted filename: RC-2022-01618.full-revision.docx [file pone.0283548.s004.docx]

**Manuscript number:** RC-2022-01618

**Corresponding author(s):** Ross, David

*[Please use this template only if the submitted manuscript should be considered by the affiliate journal as a full revision in response to the points raised by the reviewers.*

*If you wish to submit a preliminary revision with a revision plan, please use our "*[*Revision Plan*](https://docs.google.com/document/u/0/d/1HHp62Tfeb44nd3Gu_3ChB0b8gmMdEog7GjZcZFwHSiQ/edit)*" template.* ***It is important to use the appropriate template to clearly inform the editors of your intentions.****]*

## 1. General Statements [optional]

*This section is optional. Insert here any general statements you wish to make about the goal of the study or about the reviews.*

## 2. Point-by-point description of the revisions

*This section is mandatory.* *Please insert a point-by-point reply describing the revisions that were already carried out and included in the transferred manuscript.*

We thank the Reviewers for their comments. Below we have the reviewers’ comments (in blue) and our responses (in black).

Reviewer #1 (Evidence, reproducibility and clarity (Required)):

In this work, the authors claim that their machine learning approach can be combined with a biophysical model to predictably engineer sensors. The concept is interesting, but there are many issues that must be addressed before considering its publication.

1. It is surprising that their citations are too biased. They keep citing nonrelevant papers from several groups while omitting many key papers regarding genetic sensors and circuits in the field. Some can be justified (e.g., Voigt lab's reports), but others (e.g., reports on dynamic controllers too often) would not be relevant.

There are hundreds (possibly thousands) of papers that have been published on genetic sensors. Most of those papers report only qualitative results (e.g., genetic sensor implemented in a new host organism or demonstrated to sense a ligand of interest).

The purpose of this manuscript is to demonstrate methods for *quantitative* engineering of genetic sensors. Specifically, the manuscript is focused on quantitative tuning of the genetic sensor dose-response curve. So, in deciding which previous papers to cite, we chose several review articles (to cover the many, many qualitative results), any previous papers we could find that reported strategies for tuning the dose-response curve of genetic sensors (the Voigt lab’s reports and others), and any papers we could find that discussed reasons/applications for quantitative tuning of a genetic sensor dose-response curve (e.g., dynamic controllers).

We added a new paragraph to the beginning of the Results section to explain this focus on quantitative tuning (and to clearly state which statistic we use for assessing accuracy – see response to next comment; lines 72-83 in the revised manuscript).

We also added eight additional citations that we believe are relevant. We would also like to add more relevant citations as suggested by the reviewer, but that is difficult based on the reviewer’s comment, which just indicates that we have omitted many “key” papers. For the central focus of this manuscript, we think the “key” papers are those that describe methods to tune the dose-response curve of genetic sensors, and we have done our best to cite all of those that we could find. So, we ask the reviewer to please suggest some specific papers that they consider to be “key” that we should cite, or at least some more specific definition of what they think constitutes a “key” paper that should be cited.

2. It is very unclear which statistical analysis has been done for their work.

The main statistical metric used in the manuscript is the fold-accuracy. The fold-accuracy was defined in the previous version of the manuscript, but we agree that it could have been stated more clearly. So, we have moved the definition of fold-accuracy to the (new) first paragraph of the Results section, and identified it as “…the primary statistic we will use to assess different methods.” (line 77 of the revised manuscript)

3. There are many practical sensors for real applications, but their work focuses on IPTG-responsive sensors or circuits. I was wondering whether this work would have significant impacts on the field or the advancement of knowledge.

4. Similarly, it is questionable that their approach is generalizable.

Currently, there is only one published dataset that can be used for the methods described in this manuscript, for IPTG-responsive LacI variants.

However, previous work (cited in our manuscript) has shown that directed evolution can be used to qualitatively “improve” a wide range of genetic sensors beyond LacI. Furthermore, some of those previous studies used a single round of mutagenesis and libraries with diversity similar to the size of the LacI dataset (10^4^ to 10^5^ variants). Based on that, we think it is highly likely that our in silico selection approach will generalize to other sensor proteins.

With regard to the ML methods used in our manuscript, we showed in the initial publication describing the LANTERN method that the approach is generalizable to different types of proteins and protein functions (LacI sensor protein, GFP fluorescence protein, SARS Cov-2 spike-binding protein). So, we don’t see any reason to question the generalizability of that approach to other sensor proteins.

We have edited the Discussion section of the manuscript to include these points regarding the generalizability of our approach (lines 345-358 in the revised manuscript).

5. Due to the biased literature review, it is unclear to me whether this work is novel.

The majority of relevant literature on genetic sensor engineering is qualitative in nature and is not particularly comparable to the work here. We have tried to emphasize this in the introduction and discussion. We have searched the relevant literature extensively, and we have only found a small number of papers that describe quantitative methods to tune the dose-response of genetic sensors. Furthermore, there are only a few that contain any kind of quantitative assessment of that tuning. We have cited all of those papers and included specific discussions and comparisons between them and our results.

If the reviewer knows of any specific papers that we missed we would be happy to include them in our literature review.

6. I am unsure whether their correlation is sufficiently high.

This comment is too vague to address.

Again, we ask the reviewer for more specific information: What “correlation” are you referring to? And what is “sufficiently high”?

We have provided statistics on the accuracy of our methods, as discussed above.

7. Is EC50 the only important parameter? Or is it really relevant for real applications where the expression levels would change due to RBS changes, context effects, metabolic burdens, circuit topologies, etc.?

EC50 is not the only important parameter. That is why we also demonstrate the ability to quantitatively tune other aspects of the dose-response (e.g., G_∞_).

In any real application of genetic sensors, the EC50 will have to be engineered to have a quantitatively specified value (within some tolerance). So, yes, it really is relevant.

There is an important question about the effect of context however, and perhaps that is what the reviewer is really asking: If we engineer a genetic sensor that has a given EC50 in the context used for the large-scale measurement, will we be able to use that genetic sensor in a different context where, because of the change in context, its EC50 may be different?

This is one of the outstanding challenges in the field, to be able to predict the effect of a change in context. But for genetic sensors, there are several previous publications that demonstrate promising routes to quantitatively predict the effect of context on genetic sensor function.

So, we have added a paragraph to the Discussion section addressing this point and citing the relevant previous publications (lines 320-344 in the revised manuscript).

8. There are many reports on mutations or part-variants and their impacts on circuit behaviors. Those papers have not been cited. This is another omission.

As discussed in response to Comment 1, above, there are many hundreds of such papers. It would not be practical or appropriate for us to cite all of them. However, there are only a few that contain any kind of quantitative assessment of the predictability of mutational effects or of efforts to use mutations to engineer sensors to meet a quantitative specification. We have done our best to cite and discuss all of those. Again, if the reviewer knows of any specific additional papers that we should cite, please tell us.

CROSS-CONSULTATION COMMENTS

In general, I agree with the other reviewer. Its significance would be too incremental.

Reviewer #1 (Significance (Required)):

See above.

Reviewer #2 (Evidence, reproducibility and clarity (Required)):

This paper proposes two approaches for forward-design of genetically encoded biosensors. Both methods rely on a large scale dataset published earlier by the authors in Mol Syst Biol, containing ~65k lacI sequences and their measured dose response curves. One approach, termed 'in silico selection', is proposed as a way to find variants of interest according to phenotypic traits such as the dynamic range and IC50 of the biosensor dose-response curve. The second approach uses machine learning to regress the dynamic range, IC50 and others from the lacI sequences themselves - the ML regressor can then be used to predict phenotypes of new variants not present in the original dataset. The ML algorithm has been published by the same authors in a recent PNAS paper.

The manuscript has serious flaws and seems too preliminary/incremental:

1) The 'in silico selection' method corresponds to a simple lookup table. This is a perfectly acceptable method for sequence design, but the attempt to portray this as a new method or 'multiobjective optimization' is highly misleading. Also, the analogy between 'in silico selection' and darwinian evolution or directed evolution are inappropriate, because both latter approaches rely on iterative selection through fitness optimization and randomization of variants. The 'in silico selection' approach in contrast is one-shot and does not use randomization.

We agree with some of the reviewer’s points here. In making the analogy to directed evolution, we wanted to give the reader a connection to something familiar, but the reviewer is correct that the analogy is imperfect. The “lookup table” description is much better, and probably a familiar idea to most readers. So, we edited the relevant paragraph to describe in vitro selection as the use of the large-scale dataset as a lookup table instead of making the analogy to directed evolution. We thank the reviewer for this suggestion.

However, we disagree with the reviewer with regard to “multi-objective optimization.” We clearly demonstrate in Figures 3 and 4 that we can simultaneously tune multiple aspects the dose-response curve to meet quantitative specifications. If the reviewer is aware of any previous publications that they think provide a better demonstration of multi-objective engineering of biological function, please let us know; we would like to cite those papers appropriately.

Also, the reviewer is incorrect in stating that our in silico selection approach does not use randomization. The randomization occurs as part of the large-scale measurement. This is clearly stated in the second paragraph of the Results section.

2) The ML approach is a minor extension to what they already published in PNAS 2022. One could imagine an extra figure in that paper would be able to contain all ML results in this new manuscript. A couple of comments about the actual method: a) it seems unlikely to work on sequences of lengths relevant to applications, because it relies on gaussian processes that are known to scale poorly in high dimensions. b) The notion of 'interpretable ML' is misleading and quite different to what people in interpretable AI understand. Moreover, the connection between the three latent variables, which provide the 'interpretability', and biophysical models seems to come from their earlier PNAS work and this specific dataset, but there is no indication that such connection exists in other cases. Although this is somewhat acknowledged in L192-195, the text tends to portray the connection with biophysical models as something generalizable.

The ML results presented in this manuscript are specifically aimed to quantitatively assess the accuracy of the ML predictions for the parameters of a genetic sensor dose-response curve. So, we think those results belong in the current manuscript.

The reviewer’s comment on Gaussian processes and dimensionality is clearly contradicted by the results presented in this manuscript and in our previous publication describing the ML method: The ML method works quite well for “sequences of lengths relevant to applications,” including LacI (360 amino acids), the SARS-Cov2 receptor binding domain (200 amino acids), and GFP (250 amino acids). The reason for this is that the Gaussian process is only applied on the low-dimensional latent space learned by the ML method.

The reviewer’s comment on “interpretable ML” is not relevant to this manuscript but is instead a criticism aimed at our previous publication on the ML method.

The generalizability of this approach is an open question. The same could be said for most other publications describing new methods, since most of those publications include demonstrations with only a small number of specific systems. After re-reading the relevant portions of the manuscript, we disagree with the reviewer’s suggestion that we have exaggerated the potential generalizability of the approach. For example, in the last sentence of the Results paragraph, we state, “Although imperfect, this initial test of linking an interpretable, data-driven ML model to a biophysical model to engineer genetic sensors shows promise…” And, in the Discussion section, “The use of interpretable ML modeling in conjunction with a biophysical model also has the potential to become a useful engineering approach… But more rigorous methods would be needed…”

Other comments:

3) There are quite a few reduntant figures, eg Figure 1 contains too many heatmaps of the same variables. Fig 2B and C are redundant as the contain the same information. Altogether figures feel bloated and could have been compressed much more.

We disagree. The sub-panels of Figure 1 show different 2-D projections of the multi-dimensional data that are relevant to specific aspects of the results in Figs. 2-4.

Admittedly, Fig 2C shows the residuals from Fig 2B, which is in some sense the “same information.” But it is quite common, in papers focused on quantitative results, to have one sub-panel showing a comparison between predicted and actual and a second sub-panel showing the residuals.

4) Fig 2A and 3A have problems: the blue & orange lines (Fig 2A) and blue & green lines (Fig 3A) have a kink just before the second dot from the left. Such kinks cannot have been produced by a Hill function. This kind of errors cast doubt on the overall legitimacy and reproducibility of the results.

The kinks in the curves are a consequence of the use of the “symmetrical log” scale on the x-axis, which allows the zero-IPTG and non-zero-IPTG data to be shown on the same plot while showing the non-zero-IPTG data on a logarithmic scale. That symmetrical log axis uses a log scale for large x values, and a linear scale for smaller x values. The kink appears at the transition between the log and linear scales. We have re-plotted all of the figures showing dose-response curves to move the log-linear transition to overlap with the axis break.

CROSS-CONSULTATION COMMENTS

I agree with the other reviewer's comments, particularly on the lack of statistical analyses.

See our response to Reviewers #1, comment 2, above.

Reviewer #2 (Significance (Required)):

The work addresses a timely subject but is too incremental.
